# The New Wards at Newcastle Union

**Published:** 1916-02-26

**Authors:** R. Langton Cole


### The New Wards at Newcastle Union.

*To the Editor of* The Hospital.

Sir,—It would seem from the plan published in your issue of February 12 that the new wards run east and west, so that the windows on the north side receive little or no sunlight. Perhaps there is some local reason for this unusual arrangement. R. Langton Cole.

* * * The site is largely occupied by the buildings of the workhouse and its adjuncts. The objection mentioned by Mr. Cole had no substance on the day of our visit. The wards, indeed, were bright, airy, and attractive.—Ed. The Hospital.

